# Optimization of heat transfer in a double lid-driven cavity with isoperimetric heated blocks using GFEM

**DOI:** 10.1038/s41598-024-78525-w

**Published:** 2024-11-15

**Authors:** Ahmed Refaie Ali, Rashid Mahmood, Maria Ishfaq, Nusrat Rehman, Afraz Hussain Majeed

**Affiliations:** 1 Department of Mathematics and Computer Science, Faculty of Science, Menoufia University, Shebin El Kom, 32511 Menofia Egypt; 2 Department of Mathematics, Air University, PAF Complex E-9, Islamabad, 44000 Pakistan; 3 School of Energy and Power Engineering, Jiangsu University, Zhenjiang, 212013 China; 4 Department of Mathematics, Faculty of Science, New Mansoura University, New Mansoura City 35742, Egypt

**Keywords:** Mixed convection, Lid driven cavity, Non-Newtonian fluid, Isoperimetric, FEM computation, Kinetic energy, Mechanical engineering, Applied mathematics, Computational science, Fluid dynamics, Statistical physics, thermodynamics and nonlinear dynamics

## Abstract

This article is concerned with the examination of flow dynamics and heat transfer characteristics in a 1:4 double lid driven cavity in presence of isoperimetric heated blocks of various shapes. The focus is to identify the optimal shape that enhances the heat transfer in a tall cavity. The parametric settings are chosen in such a way that all the convection regimes including natural, forced and mixed convection could be generated. This cavity has lids positioned at the top and bottom, moving in opposite directions along the x-axis. The physical system is represented as a set of coupled partial differential equations incorporating the rheological properties of the power-law fluids (PL). The governing equations in conjunction with various non-dimensional physical parameters are simulated via Galerkin’s Finite Element Method (GFEM) on a very fine hybrid grid. The study includes the computation of the Kinetic Energy and Average Nusselt number to determine the optimal shape. It is concluded that the circular block is superior to the other two in terms of heat transmission efficiency.

## Introduction

Convection in cavities is a well-studied area in fluid mechanics, and there is a vast literature on this topic. Temperature variations and movements of the walls of enclosure give rise to buoyancy-driven and forced convection flow, which in turn generated mixed convection flow features. The motion of the fluid can be driven by natural convection, forced convection, or a combination of both^1,2^. The research of free convection within the cavity is among the most fundamental principles around the areas of science. The flow of the fluid and the transmission of heat qualities in different types of cavities, including square, rectangular, and circular forms, have been utilized by researchers to observe natural and forced convection. In addition to that, forced convection, which is produced on by an outside flow or a moving boundary wall, scientists have also researched on natural convection within the domain. This study has plenty of multiple applications that can be found in our daily life. For example, the phenomenon used in room ventilation and the apparatus used in cooling machines and many more.

G. DE Davis^1^ has examined laminar natural convection in a long rectangular domain, he also estimated behavior of free convection in a square air cavity having sidewall that are not heated equally^2^. S.Roy^3^ conducted an investigation on a square cavity to see the behavior in the motion of free convection due to the effect of uniformly and non-uniformly heated wall(s). Mixed convection research was done Selimefendigil and Öztop^4^ in a L-shaped wall moving domain of a nano-fluid which was done by changing the inclination angle, solid volume percentage, and Hartmann number to generate the internal heat. In a long, air-filled hollow domain with an aspect ratio of 16, Zhu and Yang^5^ quantitatively examined the emigrating natural convection for various Rayleigh numbers.In a long and double wall-driven domain the mixed convection with differential heating is researched by Öztop and Dagtekin^6^. It was discovered that the rate of heat transfer increased when the walls moved in the opposite direction. In upright chambers that have noticeably hot walls and contain air as a fluid that have variety of aspect ratios, Wakitani^7^ predicted the natural convection flow pattern. It was discovered that the essential Rayleigh number decreases due to the aspect ratio. In a tall, quadrilateral structure, Rayleigh-Bernard convection was investigated by D’Orizo et al.^8^. Latterly Lartigue^9^ explained auxiliary flow production in a long cavity of aspect ratio equals to 40, where he noted that displacement areas shift to the cavity’s descending zone at Ra = 9222. Subsequently, Basak et al.^10^ investigated mixed convection flows inside a cavity with uniform and non-uniform heating of the lower walls of the cavity.

Moreover, Javed et al. conducted an experiment in a uniformly and unevenly warm lower walls of a four-sided polygon cavity to analyze magnetohydrodynamics mixed convection. It is discovered that the enclosure exhibits stronger circulation and a dominating convection effect as the Rayleigh number rises^11^. Similarly, Lamsaadi^12^ looked at the natural convection in long quadrilateral domains for various non-Newtonian fluids and values of Prandtl from $1\le Pr\le 100$, better heat transfer rates have been observed in shear-thinning fluids. Likewise, Mendu and Das^13^ investigated forced convection for different governing parameters in a dual lid driven cavity with non-Newtonian fluids. The probability of secondary vortex production declines as the power-law index increases. In a slanted penetrable, open-ended, insignificant hollow cavity Raizah et al.^14^ computationally conducted a buoyancy-driven convection for non-Newtonian fluids. He concluded that the increase in aspect ratio reduces the amount of heat transmission rate.

Souayeh et al.^15^ conducted a numerical study on steady, laminar natural convection within a two-dimensional enclosure filled with water and containing a rectangular conducting body. Their findings demonstrate that a vertical orientation of the body results in superior heat transfer compared to a horizontal configuration. Souayeh et al.^16^ investigate the impact of varying inclination angles of a low-temperature cubical enclosure on the flow structure and heat transfer rate, with a high-temperature inner circular cylinder. Their findings reveal that the heat transfer rate increases with higher Rayleigh numbers. Souayeh et al.^17^ analyzed the impact of velocity ratio and the radius size of an inner semicircle placed at the bottom wall of a two-sided, non-facing lid-driven cavity on bifurcation phenomena. Their numerical results are integrated into a detailed correlation between the critical Reynolds number and various other parameters. Hammami et al.^18^ conduct a numerical investigation of a two-sided lid-driven cubic cavity with a cylindrical shape placed at its center. Their study reveals that as the Reynolds number increases up to 1500, the moving parallel lids generate vortices in the rear planes of the cavity behind the cylinder. Souayeh et al.^19^ conduct a systematic numerical investigation using the finite volume method combined with a full multigrid technique to study the two-dimensional and three-dimensional flow of an incompressible fluid within a cavity driven by the motion of the upper lid. Their findings conclude that a bidimensional cavity with an inner circle at the center exhibits a higher critical Reynolds number compared to one without the inner circle, leading to a delay in flow unsteadiness.

While referring to K.M. Gangawane’s work^20^, an investigation was conducted utilizing numerical techniques resulting from mixed convection in a quadrilateral domain to examine the dynamics of transfer of heat and flow of the fluid. In this study, motion was imparted to one of the vertical walls, while a centrally positioned elliptical block was heated to maintain a stable condition. The primary aim of this research was to investigate the impacts of various flow parameters, for instance the vertical wall’s direction of motion (either up or down along the y-coordinate axis) and the elliptical cylinder’s aspect ratio (0.5, 1, 2). Numerous dimensionless numbers were investigated, including *Gr *$(1-100)$*, Pr* $(1-100)$, and *Re* $(1-5000)$, respectively. The finite volume technique (FVM) and the SIMPLE numerical methodology were used in the simulations. To acquire a complete knowledge of the enclosure’s physical behavior, the inquiry included a rigorous assessment of streamline and isotherm profiles. Additionally, the study calculated the Nusselt number (Nu) for circular shape and elliptical cylinder and compared the average rate of heat transmission of both the cases. The findings exhibit that the rate of thermal transmission has a more substantial effect due to the motion of the walls as compared to the size of the elliptical cylinder. Furthermore, it was observed that the cavity which contains a vertically upward moving wall exhibited an enhanced heat transfer rate. The domain with a heated elliptical block (Er ≠ 1) has high rate of thermal transmission convection in comparison with the one which has a purely circular shape.

Elboughdiri et al.^21^ visualized thermal energy within an Oldroyd-B fluid model by incorporating different types of nanoparticles to enhance thermal performance. Their findings indicate that increasing Eckert number, magnetic number, solar thermal radiation number, and electric field number leads to improved temperature profiles. Sohail et al.^22^ explored the application of a tetra-hybrid nanofluid (containing aluminum oxide, iron dioxide, titanium dioxide, and copper) in a crossflow model over a vertical disk. Their study considered the impact of nanoparticle shapes (bricks, cylindrical, and platelets), the influence of electro-magneto-hydrodynamic effects, and the effect of quadratic thermal radiation. Their findings suggest that tetra-hybrid nanofluids offer a significant advantage in industrial applications demanding the highest thermal energy production. Elboughdiri et al.^23^ conducted a mathematical analysis of dual simulations involving tangent hyperbolic rheology, heat transfer, and mass diffusion on an expanding/shrinking needle. Incorporating Darcy’s Forchheimer law and a magnetic field, their study revealed that increasing chemical and Schmidt numbers led to a decrease in concentration profiles. Sohail et al.,^24^ implemented finite element method to simulate the hybrid nanofluid model in a Riga plate and compared the thermal performance of the system.

Numerical analysis is conducted in a wall moving domain by Billah, M. M., et al. ^25^ to investigate the phenomenon of thermal transmission due to mixed convection, where a hot empty cylinder is placed in the middle of the cavity. Billah, M. M., et al. Conducted this study to simulate realistic systems such as ovens that have heaters and air-conditioned electrical equipment. To solve the governing equations, a Newton–Raphson algorithm combined with a Galerkin’s weighted residual finite scheme is utilized. The calculations encompass a broad spectrum of ratio of thermal conductive solids and fluids, diameters of cylinders and Richardson numbers The findings are shown through the pictorial representation of average Nusselt number, temperature contours, velocity profiles and streamlines on the heated surface, and different specified parameters are considered in the domain of thermally distributive fluid. It is noted that substantial sensitivity to the diameter of a cylinder as well as the ratio of heat conductivity of solid fluid have been displayed by the distribution of temperature and field of flow in the three convective regimes. Islam et al. have quantitatively examined the features of a square obstruction which is heated at equilibrium temperature to analyze the properties of mixed convection inside a four-sided closed domain. The cavity consists of obstructions that are located at its upper left and lower right edges to provide the best heat transmission results which is determined by the Nusselt number^26^. Numerical results for non-Newtonian fluids in a double, wall-moving quadrilateral domain with a decentered heated triangular obstacle having a constant temperature, have been presented by Manchanda and Gangawane^27^. For fluids with n = 0.2, it was discovered that the impact of the mixed convection variables had little bearing on the fluid and thermal structure inside the cavity. Vijayan and Gangawane^28^ did computational study to investigate the movement of fluids and transfer of heat characteristics resulting from combination of forced and free convection in a long wall moving domain holding an equally heated equilateral triangular block. The division of fluid and its structural behavior in the cavity becomes increasingly erratic as the aspect ratio (AR) of the enclosure increases. Thus, when the aspect ratio of the considered space grows larger, the distribution of fluid and its structural behavior within the cavity becomes more scattered. In a study by Cheng et al.^29^, the authors investigated the features of heat transfer through mixed convection in a wall moving square cavity, considering different Richardson and Prandtl numbers. The researchers present a temporal analysis of the kinetic energy and average Nusselt number, demonstrating the progression from laminar to chaotic flows. The experiments were conducted with a horizontal orientation, Laidoudi and Ameur^30^ quantitatively investigated mixed convection between two revolving circular cylinders. The outcomes for various Prandtl-Richardson numbers, fluid index, and Reynold values have also been depicted. In a two-dimensional hollow having a sliding lid, mixed (combined) convective non-Newtonian fluid flow is numerically simulated by Thoura et al. in^31^. where he discovered that the standard deviation of thermal transmission rate decreases as the Richardson number is incremented for a particular skew angle. Using a fillet square cavity as a model, Rehman et al.^32^ performed a numerical analysis of a magnetized ferric oxide–water nanofluid. It can be inferred that the fluid flows over the cylinder as it revolves in a clockwise direction. Furthermore, increasing ferroparticle volume and angular velocity increases Nusselt number and reduces thermal and viscous entropy. Similarly, Safaei.et al.^33^ conducted a mathematical investigation on a square domain with cold right and left walls, an adiabatic upper wall in motion, and a heated bottom wall held fixed. It is discovered that the isothermal lines, which govern natural convection, are essentially symmetric, and that as forced convection progresses, these lines become asymmetric. In a discretely heated wall-driven domain, Roy et al.^34^ explored different power-law fluids along with their thermal features. According to this study, the greater the power-law index, the more effectively driven and natural mixed convection transport heat. Furthermore, if the Reynolds or Grashof numbers are held constant while changing the Richardson number, the efficiency of heat transmission changes. Pure mixed convection in lid driven enclosure has been simulated by Chowdhury et al.^35^. They considered the power-law model.

Moreover, Ali, I. R.^36^ utilized a hybrid nanofluid consisting of $\text{Al}2\text{O}3-\text{Cu}-$ Water in a double moving wall quadrilateral enclosure to improve the transmission of heat during this investigation he employed Finite Volume Method (FVM) for results. The SIMPLE algorithm is used to address the pressure–velocity coupling. The results show that using hybrid nanofluid in a tall enclosure significantly improves heat transmission. Additionally, it is observed that a larger solid body can further improve heat transfer at higher Reynolds numbers. Thermal transmission is found to increase with increasing Richardson number. In relation to that Gangawane^37^ investigates the impact of the position of a heated triangular obstacle positioned along the vertical centerline of a top wall moving square domain on the properties of mixed convection. The analysis considers a 2D, laminar, time independent flow of a Newtonian and incompressible fluid. The cavity’s upper and lower walls are considered adiabatic, whereas the left and right walls have an ambient temperature (Tc). Hossain^38^ examines a 2D mixed convection flow of an incompressible and viscous fluid with viscosity depending upon the temperature past an impermeable upright surface. Perturbation techniques are utilized to solve the reduced equations in the forced and free convection regimes. The numerical outcomes are shown in relation to the local edge heat flux and shear stress.

In recent studies, various numerical and computational techniques have been employed to enhance the understanding of fluid dynamics and related fields. For example, Refaie Ali et al.^42^ investigated the enhancement of hydrodynamic forces through miniaturized control of square cylinders using the lattice Boltzmann method. Additionally, another study by Refaie Ali et al.^43^ presented an AI-based predictive approach using CFD-ANN and Levenberg–Marquardt in the context of a driven-cavity of Ostwald de-Waele fluid. Furthermore, Majeed et al.^44^ conducted numerical simulations to evaluate energy storage performance in a close configuration using Galerkin finite element-based computation. Studies like these, along with others focusing on fluid–structure interactions^45^, fractional differentiation applications^46^, and the properties of new statistical distributions^47^, continue to contribute significantly to the field. Research on electrohydrodynamic stability^48^, heat and mass transfer in nanofluid flows^49^, and the effects of magnetic fields on bio-viscosity flows^50^ also provide valuable insights. Recent work has further explored the influence of magnetic fields on mixed convection in fluid systems^52^, and symmetry-based analyses of nonlinear mixed convection^51^ have advanced the understanding of multi-physical interactions in complex fluid flows. Additionally, research by Al-Showaikh^53^ on the effects of non-uniform geometry on peristaltic flow offers new perspectives on fluid dynamics in biomedical applications.

The examination of mixed convection that takes place in a rectangular cavity is the main topic of the current work. The extension from the available studies deals with the presence of three different iso-perimetric shaped heated blocks of circular, square and triangular shape. The objective of the current article is to estimate the effects of different iso-perimetric shapes on heat transfer rates and convection process.

## Problem description

**Fig. 1:**
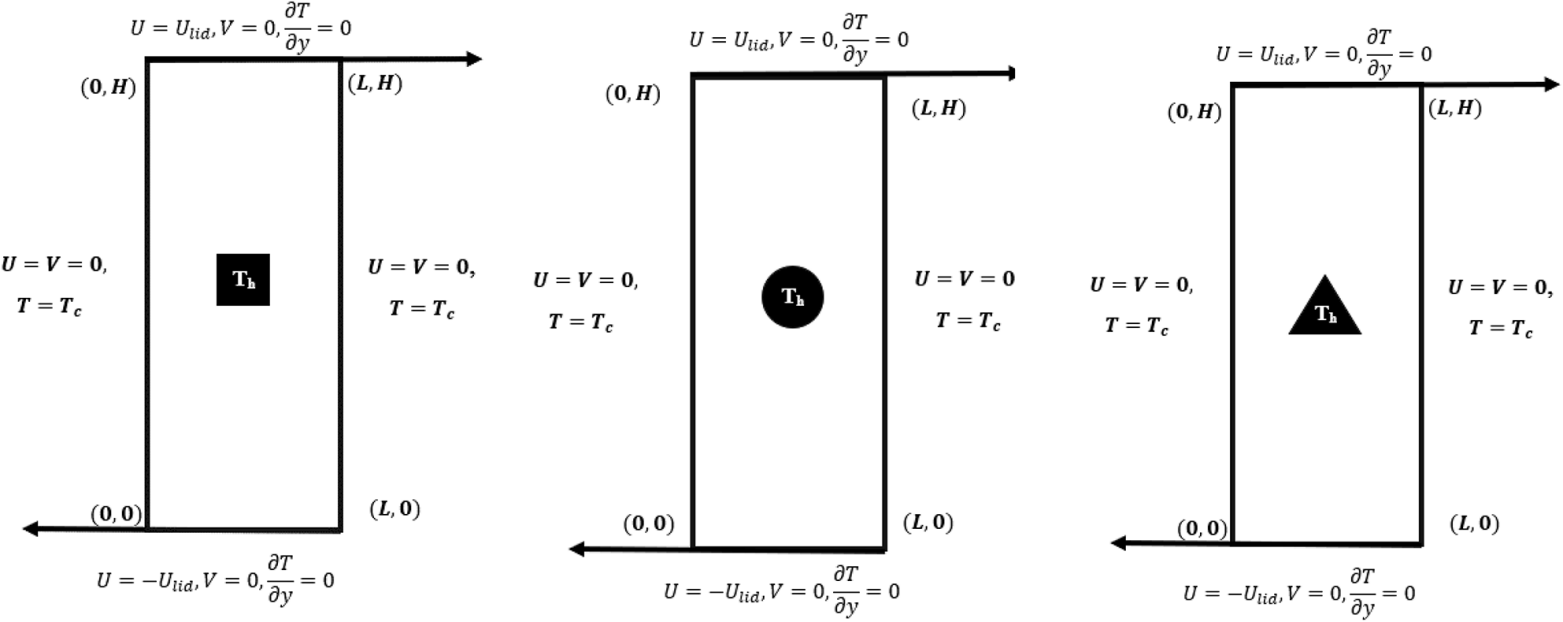
Schematic diagram of isoperimetric heated blocks.

A steady, incompressible, and laminar mixed convective flow and thermal transmission in a 2D rectangular domain of length $L$ and height $H$ is considered respectively. A heated block submerged at the geometric center of the domain is maintained at a constant temperature ${T}_{H}$. Figure 1 depicts the physical properties and cartesian coordinate system considered during the inquiry. In Fig. 1, the space coordinates $(x, y)$ and related velocity components $(u, v)$ are also shown. The cavity’s top and bottom walls are permitted to move at the same velocity ${U}_{lid}$ but in opposing directions. The cavity’s horizontal walls are thermally insulated. A constant ambient temperature $({T}_{c}{<T}_{H})$ is maintained along the cavity’s left and right vertical walls. Calculating the density fluctuation with temperature makes use of the Boussinesq approximation. Radiation and viscous thermal dissipation have been considered to have insignificant effects on heat transmission^39^. Temperature independence is assumed for all the thermophysical characteristics. Consider three shapes Triangle, Circle and Square of same perimeter as heated blocks. As we all know that the perimeter of circle is equal to circumference of the circle which is given as $2\pi r$, where $r$ is the radius of the circle. And the perimeter of Square is acquired as $4x$, where x is the length of each side. Considering the perimeter of triangle fixed, we have computed the values of $r$ and $x$ and obtained two new cases depicted in Fig. 1.

## Mathematical formulation & benchmark quantities

Based on the assumptions stated in previous section, following dimensional governing equations govern the flow dynamics in the cavity:

1 $$\frac{\partial u}{\partial x}+\frac{\partial v}{\partial y}=0$$

2 $$\rho \left[ u\frac{\partial u}{\partial x} +v \frac{\partial u}{\partial y} \right]+\frac{\partial p}{\partial x}=\left(\frac{\partial {\tau }_{xx}}{\partial x}+\frac{\partial {\tau }_{xy}}{\partial y}\right)$$

3 $$\rho \left[ u\frac{\partial v}{\partial x} +v \frac{\partial v}{\partial y} \right]+\frac{\partial p}{\partial y}=\left(\frac{\partial {\tau }_{xy}}{\partial x}+\frac{\partial {\tau }_{yy}}{\partial y}\right)+\rho \beta g(T-{T}_{C})$$

4 $$\rho {c}_{p}\left[u\frac{\partial T}{\partial x} +v\frac{\partial T}{\partial y} \right]=k\left[\frac{{\partial }^{2}T}{\partial {x}^{2}}+ \frac{{\partial }^{2}T}{\partial {y}^{2}} \right]$$

where ${\tau }_{ij}$ is the stress component, ${\tau }_{ij}=2\mu \dot{(\gamma )}{\varepsilon }_{ij}$

And ${\varepsilon }_{ij}$ is the deformation tensor given as ${\varepsilon }_{ij}=\frac{1}{2}\left(\frac{\partial {u}_{i}}{\partial {x}_{j}}+\frac{\partial {u}_{j}}{\partial {x}_{i}}\right)$, $\beta$ shows the coefficient of thermal expansion,${c}_{p}$ represents specific heat and $k$ exhibits thermal conductivity, respectively.

Since the model presented in Eqs. (1–4) is non-linear and coupled and each quantity in these equations has its own dimensions. To obtain dimensionless set of equations, we use the following scaling transformations

$$\overline{X }\text{ = }\frac{x}{H}$$, $$\overline{Y}\text{ } = \frac{y}{H}$$, $$\overline{U }\text{ = }\frac{u}{{U}_{lid}}$$ , $$\overline{V}\text{ } = \frac{v}{{U}_{lid}}$$ , $$\overline{P }\text{ = }\frac{p}{{\rho U}_{lid}^{2}}$$

$${\theta = }\frac{T-{T}_{C}}{{T}_{H}-{T}_{C}}$$

The coordinates, components of velocity, pressure, and temperature are all represented in the context by the dimensionless variables $\overline{X }$, $\overline{Y }, \, \overline{U }, \, \overline{V } \, ,\overline{P }$ and ${\theta}$ respectively. Putting and arranging the above values in Eqs. (1–4), the following non-dimensional form is obtained:

5 $$\frac{\partial \overline{U} }{\partial \overline{X} }+\frac{\partial \overline{V} }{\partial \overline{Y} }=0$$

6 $$\left[\overline{U }\frac{\partial \overline{U} }{\partial \overline{X} } +V \frac{\partial \overline{U} }{\partial \overline{Y} } \right]+\frac{\partial \overline{P} }{\partial \overline{X} }=\frac{1}{Re}\left( 2\frac{\partial }{\partial \overline{X} }\left(\frac{\eta }{m}\frac{\partial \overline{U} }{\partial \overline{X} }\right)+\frac{\partial }{\partial \overline{Y} }\left(\frac{\eta }{m}\left(\frac{\partial \overline{U} }{\partial \overline{Y} }+\frac{\partial \overline{V} }{\partial \overline{X} }\right)\right)\right)$$

7 $$\left[\overline{U } \frac{\partial \overline{V} }{\partial \overline{X} } +\overline{V } \frac{\partial \overline{V} }{\partial \overline{Y} } \right]+\frac{\partial \overline{P} }{\partial \overline{Y} }=\frac{1}{Re}\left( 2\frac{\partial }{\partial \overline{Y} }\left(\frac{\eta }{m}\frac{\partial \overline{V} }{\partial \overline{Y} }\right)+\frac{\partial }{\partial \overline{X} }\left(\frac{\eta }{m}\left(\frac{\partial \overline{U} }{\partial \overline{Y} }+\frac{\partial \overline{V} }{\partial \overline{X} }\right)\right)\right)+Ri\times \theta$$

8 $$\left[\overline{U} \frac{\partial \theta }{\partial \overline{X} } +\overline{V} \frac{\partial \theta }{\partial \overline{Y} } \right]=\frac{1}{Pr\times Re}\left[\frac{{\partial }^{2}\theta }{\partial {\overline{X} }^{2}}+ \frac{{\partial }^{2}\theta }{\partial {\overline{Y} }^{2}}\right]$$

where $\eta$ is the apparent viscosity given as.

$$\eta =m{\left\{2\left[{\left(\frac{\partial \overline{U} }{\partial \overline{X} }\right)}^{2}+{\left(\frac{\partial \overline{V} }{\partial \overline{Y} }\right)}^{2}\right]+{\left[\left(\frac{\partial \overline{U} }{\partial \overline{Y} }\right)+\left(\frac{\partial \overline{V} }{\partial \overline{X} }\right)\right]}^{2}\right\}}^{\frac{(n-1)}{2}}$$

And the parameters $m$ and $n$ are representing the consistency parameters and power-law indices, respectively. Here $Ri=\frac{Gr}{{Re}^{2}}$ is the Richardson number. The generalized expressions for *Pr*, *Re* and *Gr* can be found in^35,36^. These expressions reduce to their classical forms for *n* = *1*.

### Local Nusselt number

The local Nusselt number represents the heat transfer coefficient at a specific point on a surface or within a flow. It is given by the formula:

$${Nu}_{Local}=\frac{h\left(x\right).\mathcal{L}(x)}{\mathcal{K}}$$

where $h\left(x\right)$ is the local heat transfer coefficient, $\mathcal{L}(x)$ is the characteristic length at the location *x*, and $\mathcal{K}$ is the thermal conductivity of the fluid.

### Average Nusselt number

The average Nusselt number is calculated by averaging the local Nusselt numbers over a specified area or length. It provides an overall measure of heat transfer performance for the entire surface or flow. The formula is:

$${Nu}_{avg}=\frac{1}{\mathcal{L}}\underset{0}{\overset{\mathcal{L}}{\int }}{Nu}_{Local}\left(x\right)dx$$

where $\mathcal{L}$ is the total length or area over which the average is computed, and ${Nu}_{Local}(x)$ is the local Nusselt number at position *x*.

The kinetic energy (KE) is another global secondary quantity that evaluates the scale of momentum for entire flow. The kinetic energy is defined as

$$KE = \frac{1}{2}\int\limits_{\Omega } {\left\langle {u.u} \right\rangle d\Omega .}$$

## Solution approach & solvers

For solving the model presented in Eqs. (5)-(8), we have employed Finite Element Method based commercial software COMSOL 5.6 which offer Taylor–Hood family of stable finite elements for the approximation of velocity, temperature and pressure approximations. Moreover, a library offering nonlinear and linear solvers is also available. In the FEM procedure, the physical domain is discretized into a limited number of elements, appropriate element shapes and computation functions are chosen, the elemental equations are put together into an overall set of equations, and these equations are then solved using numerical techniques^40,41^. The fundamental discrete nonlinear system of equations was linearized by using the well-known Newton’s technique, and then the resulting linearized inner systems were solved with an exact solver named PARDISO that actually uses the method relies on breaking down matrices into lower and upper triangular matrices commonly known as “LU matrix factorization”, which diminishes the number of iterations mandatory to achieve the desired level of accuracy or convergence.

**Fig. 2:**
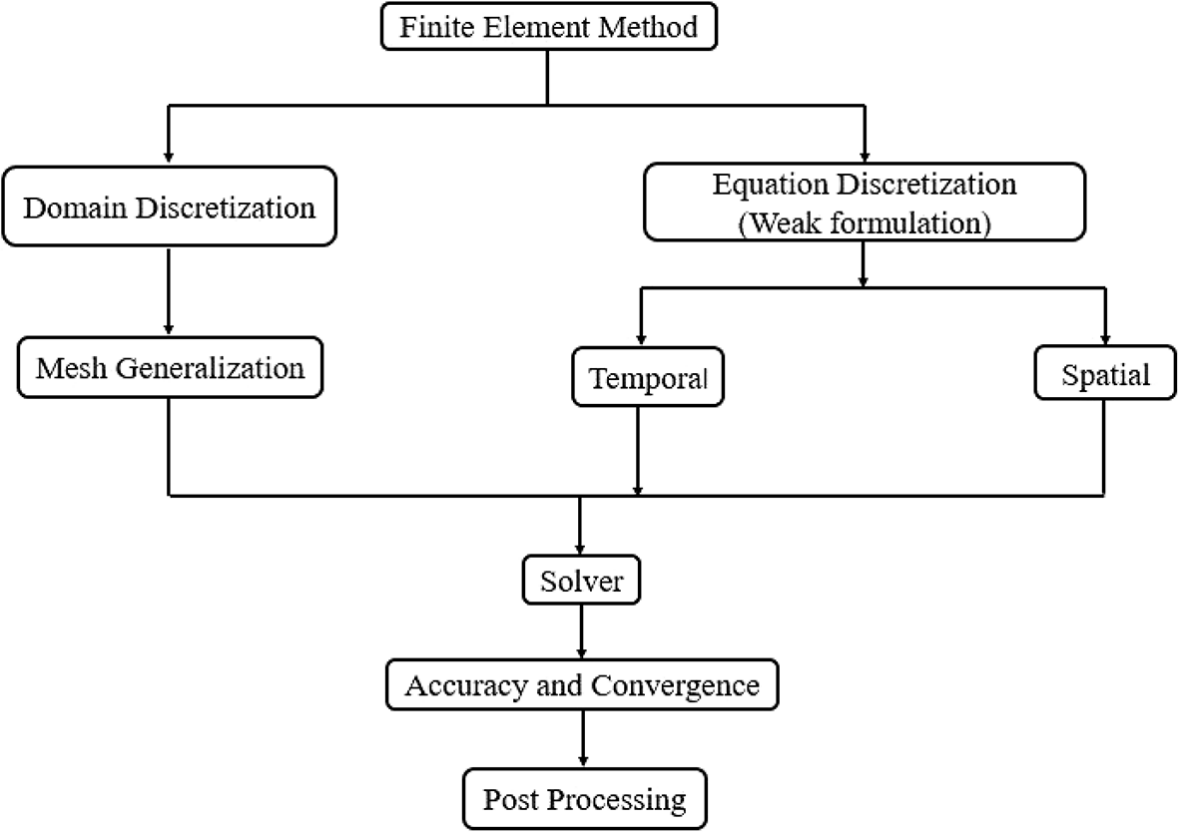
Flow diagram of finite element method.

The flow chart representing the finite element method (FEM) is illustrated in Fig.2 .

**Fig. 3:**
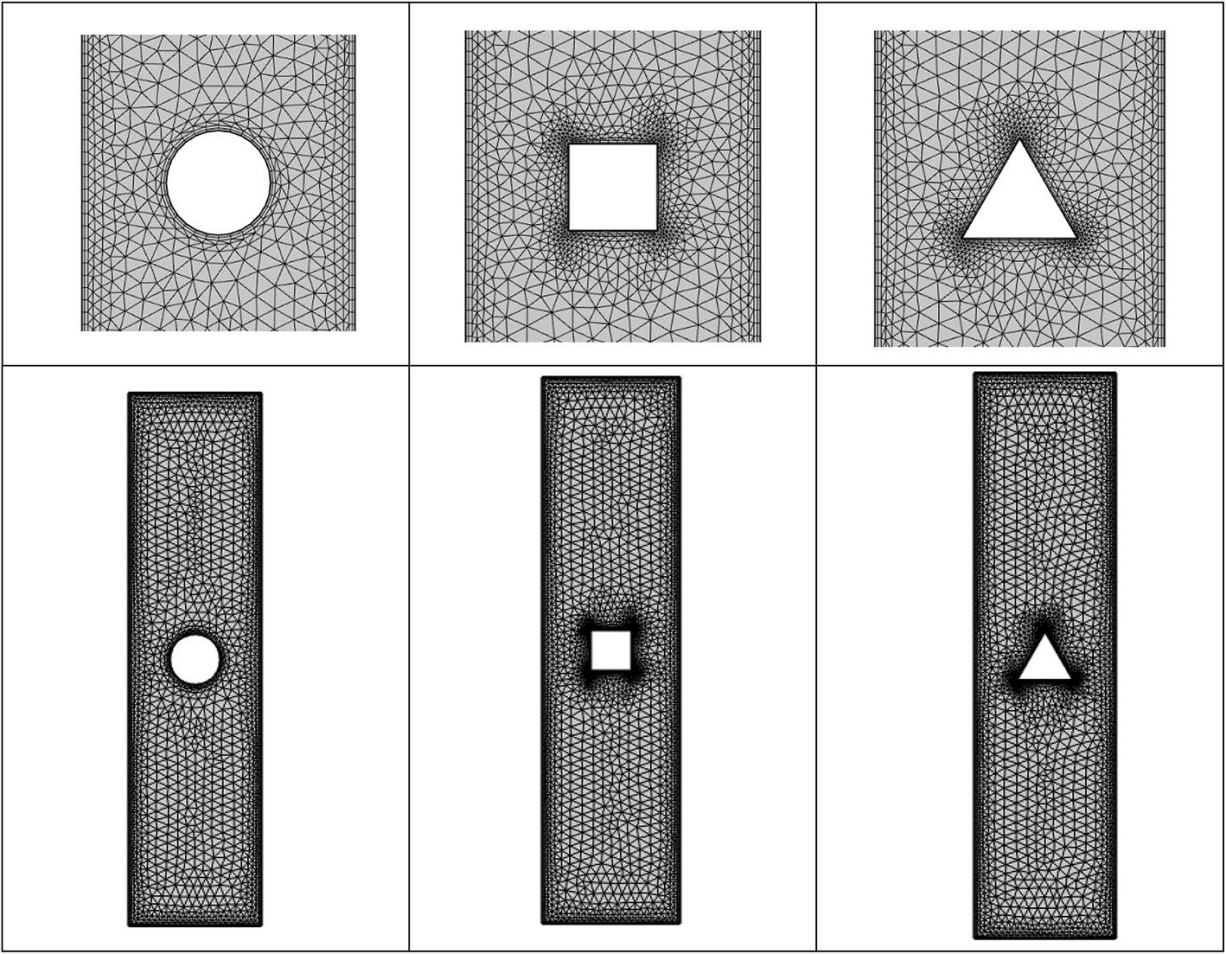
Coarse refinement level grid.

Figure 3 depicts the coarse computational grid; to show the flow dynamics at the boundaries properly, we have utilized hybrid meshing for better and more accurate results.

A grid independence test was conducted using four grid sizes, as detailed in Table 1. The parameter ${Nu}_{\text{avg}}$ was utilized to quantify the test and the study was performed with *Pr* = 10, and *Ri* = 1 for the circular block case. This configuration ensures that the deviation of selected quantities between grid levels extra fine and extremely fine is negligibly small. Consequently, simulations are executed at extra fine level to optimize computational resources. The table includes the corresponding number of elements (#EL) and total unknowns or degrees of freedom (#DOFs). Table 2 shows the refinement levels, number of elements, and degrees of freedom. The table also shows that for all cases of iso-perimetric blocks namely circle, square, and triangle we have more than 200,000 degrees of freedom (at Extremely fine refinement level). Thus, we can say that they have comparable degrees of freedom for the comparison of results and drawing conclusions regarding the optimal configuration.

**Table 1:** Grid convergence test for the given problem Pr = 10 and Ri = 1.

Levels	#EL	#DOF	${Nu}_{\text{avg}}$	% deviation
Normal	5428	12,485	5.137069	–
Fine	9156	20,331	5.152013	0.29
Finer	22,936	50,191	5.191294	0.76
Extra fine	57,026	122,431	5.208933	0.33
Extremely fine	96,252	210,883	5.208293	0.01

**Table 2:** Refinement levels for different iso-perimetric shapes.

Refinement level	Square block		Circular block		Triangular block	
	$\#EL$	$\#DOF$	$\#EL$	$\#DOF$	$\#EL$	$\#DOF$
Extremely Coarse	976	2439	776	2011	930	2339
Extra coarse	1572	3861	1276	3221	1536	3786
Coarser	2476	5899	1946	4751	2432	5793
Coarse	4470	10,337	3548	8365	4340	10,054
Normal	6694	15,165	5428	12,485	6562	14,878
Fine	11,364	24,955	9156	20,331	10,962	24,103
Finer	26,322	57,251	22,936	50,191	26,072	56,683
Extra fine	63,626	136,119	57,026	122,431	62,666	134,066
Extremely fine	104,338	217,543	96,252	210,883	103,214	215,162

Table 3 illustrates the comparison between the current simulation results and those reported by Kumar et al.^39^, who studied mixed convection within a lid-driven cavity containing a triangular block. A detailed examination of Table 3, with the same geometric and parametric settings used in this study yields accurate results**.**

**Table 3:** Code validation study: comparison of ${Nu}_{\text{avg}}$ with Kumar et al.^39^.

*Ri*	Current work	Kumar et al.^39^
0.1	4.600314564213044	4.7241
1	4.600316485541422	4.7157
10	4.600316759670698	4.7151

## Results and discussions

Through the examination of streamlines and velocity profiles, the convective behavior and properties within the rectangular cavity were investigated, considering the influence of various parameters. These parameters include the Richardson number $(0.1 \le Ri \le 10)$, non-Newtonian fluids $(0.6 \le n \le 1.4),$ aspect ratio of $0.25$ and Prandtl number from $(1, 10)$.

The objective of the current numerical simulation was to examine mixed convection in a long, double-wall moving domain that was governed by a lid and comprised three distinct iso-perimetric-shaped blocks that were heated evenly. Numerous parameters, including the coefficient power-law and the Richardson number $(0.1, \text{1,10})$, have been seen to have an impact.

The effects of the index $n=(0.6, 1, 1.4)$, Pr $(1, 10),$ and $\text{AR }= 0.25$ on the convective characteristics of the long rectangular cavity have all been investigated. Thermal contours and streamlines are used to examine the convection properties. As viscosity increases and the Prandtl number approaches $Pr = 10$, momentum diffuses more quickly than heat. The isotherm profiles shown in the figure show the combined effects of the moving wall’s motion and the convection currents created by buoyancy travelling between the heated blocks and walls. The increase in the power-law index corresponds to an increase in Nu values when $AR = 0.25$ and all other factors are considered.

The two famous dimensionless parameters, Nusselt number (Nu) and Richardson number (Ri), are commonly used in fluid mechanics to describe the characteristics of fluid flow and heat transport. A kind of fluid phenomenon called “mixed convection” mixes free convection with forced convection. The value of the Nusselt number can be influenced by the Richardson number as well as other dimensionless variables like Re and Pr. Specifically, for mixed convection in a rectangular domain, Nu rises as the Ri rises.

Prandtl number also has a highly special relationship with Nu that is crucial in determining the characteristics of heat loss and fluid dynamics. According to their relationship, raising Pr for a given fluid and a certain flow state would result in a rise in the Nusselt number, respectively.

### Impact of parameters on velocity profiles:

This section represents the velocity profiles for all three iso-perimetric shaped heated blocks for Aspect Ratio 0.25 and fixed Prandtl number Pr = 1, while other parameters like non-Newtonian fluid index vary from $0.6\text{ to }1.4$ and Richardson numbers change from 0.1 to 10.

**Fig. 4:**
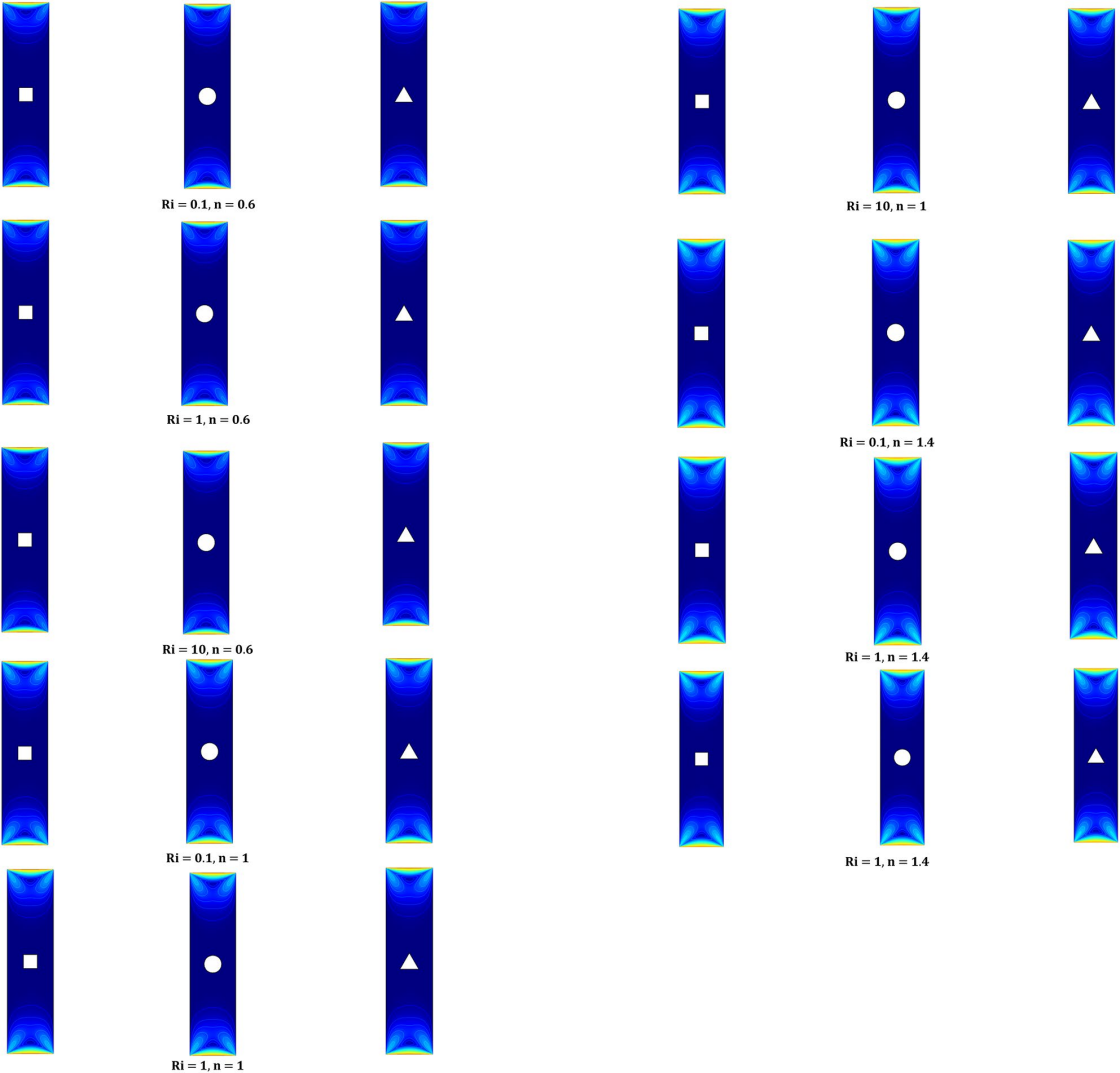
Variation on velocity profiles for the *Ri* (0.1,1,10), *Pr* = 1 and *n* (0.6,1,1.4).

Figure 4 shows the impact on the velocity of fluid due to the varying values of *n* and *Ri* for different iso-perimetric shaped heated blocks, The velocity is maximum at the lower and upper ends of the cavity due to the no slip boundary condition which is decreases as we move towards the center. Through figure we can also observe that when we increase the indices between $0.6\text{ to }1.4$ (from shear thinning to shear thickening) the size and strength of the vortices increases. We do not see any significant change in the velocity profiles by increasing the values of Ri from $0.1\text{ to }10$.

### Impact of parameters on streamlines

Streamline contours are shown in this section for the three iso-perimetric shaped heated blocks for AR 0.25, with various parameters like power-law index n, *Ri* and *Pr,* respectively.

**Fig. 5:**
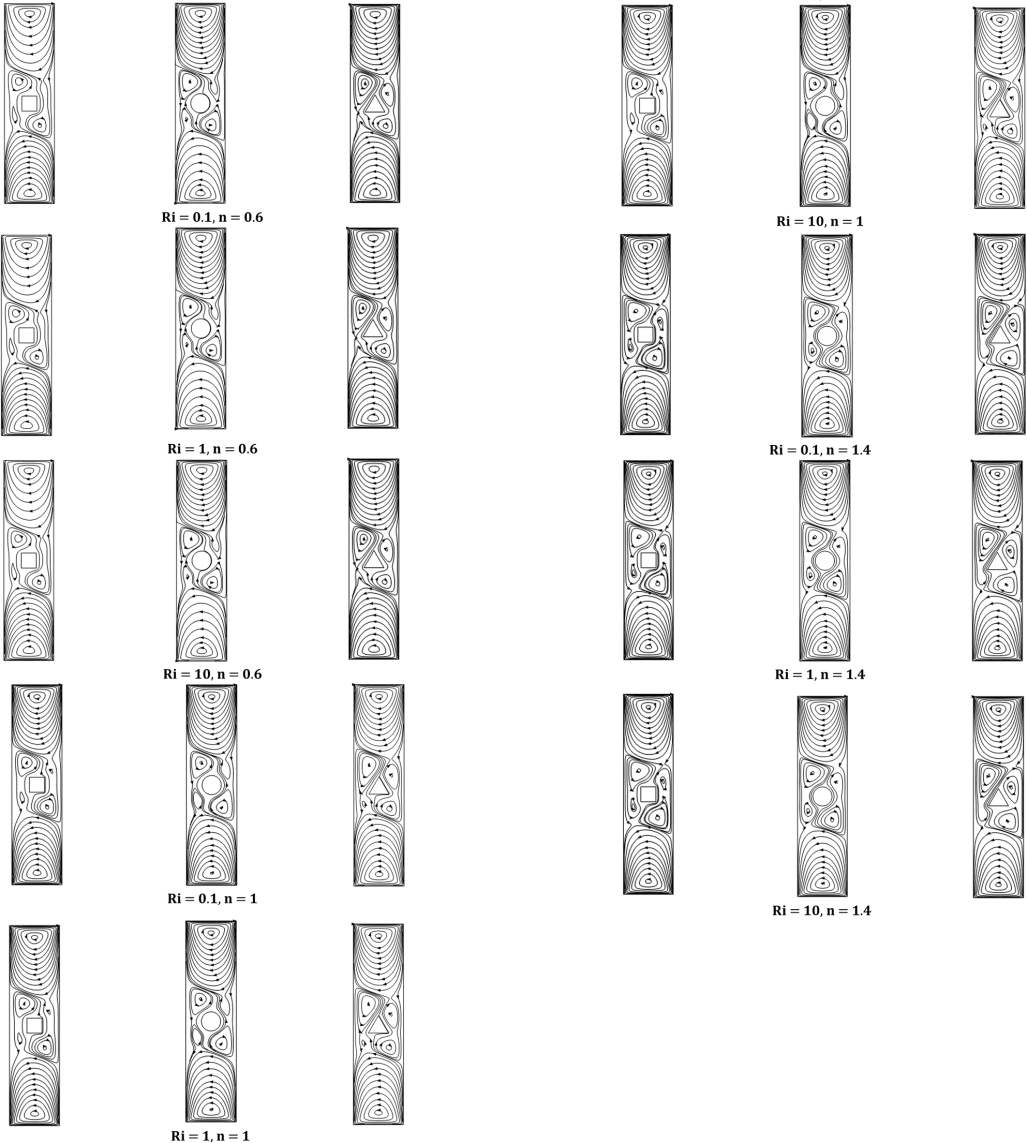
Variation on streamlines for the *Ri* (0.1,1,10), *Pr* = 1, and *n* (0.6,1,1.4).

Streamlines are represented by Fig. 5 for fixed *Pr* = 1 and AR = 0.25 along with different varying parameters (Ri and n). The motion of the lids generates primary vortices at the bottom and top ends of the domain in clockwise and anticlockwise directions, while the obstacle creates secondary vortices. Figure 5 depicts that the strength, size, and quantity of secondary vortices increase along with the values of *Ri* and *n*.

When studying the flow behavior in mixed convection, where both forced and natural forces play a significant role, the Ri is frequently utilized. In mixed convection, buoyancy and shear, both of which are frequently impacted by the kinetic energy of the fluid, drive the fluid motion. *Ri* and kinetic energy have no direct relationship, but the fluid’s kinetic energy can influence the fluid’s temperature dispersion and the related temperature gradient.

Table 4, 5, 6 represents the values of ${Nu}_{\text{avg}}$ for all three cases of iso-perimetric shaped heated blocks against varying parameters. For circles, Square and Triangles for all these cases ${Nu}_{\text{avg}}$ progressively increases with the enhancement in the values of *Ri* and *n.* And ${Nu}_{\text{avg}}$ also shows an increasing trend with *Pr* from 1 to 10, respectively.

**Table 4:** Variation in ${Nu}_{\text{avg}}$ for triangular block.

Triangular block			
*Ri*	$\text{n}=0.6$	$\text{n}=1$	$\text{n}=1.4$
*Pr* = 1			
0.1	4.6003126209978245	4.600314564213044	4.60031571068493
1	4.600313717169332	4.600316485541422	4.600323225269491
10	4.600313717610603	4.600316759670698	4.6003232596343775
*Pr* = 10			
0.1	4.600359754877665	4.600388687606466	4.600588461864127
1	4.600360840253299	4.600458619229786	4.600605981426356
10	4.600360840634940	4.600458893474137	4.600606015736777

**Table 5:** Variation in ${Nu}_{\text{avg}}$ for circular block.

Circular block			
*Ri*	$n=0.6$	$n=1$	$n=1.4$
Pr = 1			
0.1	5.2080635250405685	5.207986205207084	5.2080468930227015
1	5.208064765973295	5.208065363577031	5.20806672138569
10	5.208064766563796	5.20806567400318	5.2080667602122
Pr = 10			
0.1	5.208748196021137	5.208758196513681	5.208922582846382
1	5.208749424643913	5.208837373703201	5.2089424174913574
10	5.208749425050214	5.208837684202272	5.208942456322602

**Table 6:** Variation in ${Nu}_{\text{avg}}$ for square block.

Square block			
*Ri*	$n=0.6$	$n=1$	$n=1.4$
Pr = 1			
0.1	4.811289407970605	4.811297930522045	4.811299925365667
1	4.81129055434863	4.811291058268757	4.811302243126531
10	4.811290554958135	4.811291345052082	4.811302278997555
Pr = 10			
0.1	4.812014451849504	4.812019103903576	4.812165304403382
1	4.812015587491476	4.8120922503959225	4.8121836286717805
10	4.812015587901151	4.812092537252813	4.812183664481747

In Table 4, 5, 6, the circular heated block has the greatest influence on ${Nu}_{\text{avg}}$ when compared to the other iso-perimetric shapes.

Table 7, 8, 9 presents the values of KE for a range of parameters. The KE for all three iso-perimetric shaped heated blocks have been represented for different values of *Ri*, *n,* and *Pr* from 1 to 10. The table shows that while keeping other parameters constant, increasing the Pr from $1\text{ to }10$ improves the thermal transmission and certainly the Kinetic energy. With the rise in the values of fluid’s consistency index from $\text{n}=0.6\text{ to }1.4$, values of KE significantly increase.

**Table 7:** Variation in $KE$ for triangular block.

Triangular block			
*Ri*	$n=0.6$	$n=1$	$n=1.4$
Pr = 1			
0.1	0.04720643724518969	0.0675661303065068	0.08057750810305506
1	0.047206885310719325	0.0675681842542786	0.08057812227043229
10	0.047206889187151933	0.06756819230903285	0.08057812347193449
Pr = 10			
0.1	0.047206143587818286	0.06756569227419969	0.08057768096276772
1	0.04720889169405125	0.06756774619644185	0.08057869513091456
10	0.04721090303598736	0.06756775425109507	0.08057869633242241

**Table 8:** Variation in $KE$ for circular block.

Circular block			
$Ri$	$n=0.6$	$n=1$	$n=1.4$
Pr = 1			
0.1	0.04725910551169377	0.06757649679653465	0.08058264400484288
1	0.04729984570024966	0.0675785507563644	0.08058525818674172
10	0.04729987440851826	0.06757855881115656	0.08058995938827212
Pr = 10			
0.1	0.0473880652011413	0.067593429633011186	0.08059864810662241
1	0.04738822553449027	0.06759634931649592	0.0805992619679327
10	0.04738872451466285	0.06759935736749064	0.08059996316880193

**Table 9:** Variation in $KE$ for square block.

Square block			
$Ri$	$n=0.6$	$n=1$	$n=1.4$
Pr = 1			
0.1	0.04720736281531378	0.06756753988364314	0.08057892870481691
1	0.04721210599811218	0.06756959387455652	0.08057954289574767
10	0.0472150044599164	0.0675696019294787	0.08057960109731186
Pr = 10			
0.1	0.047280436261529665	0.06757097815576746	0.08058025762622526
1	0.04729519518174297	0.06757303103577754	0.08058087146748723
10	0.047305094120620334	0.06757313908635284	0.08058088266834992

As observed, changing the shape of the heated block affects how rapidly heat is transferred, with circular shape having the most effect. It may be the result of the fluid surrounding a heated circular block becoming buoyant due to the temperature differential. This natural molecular force can cause the fluid to go upward or downward depending on the position, shape and size of the block and the direction of the temperature gradient, which can lead to complex fluid motion around the block.

## Conclusion

Three iso-perimetric but various-shaped obstacles have been placed in a rectangular enclosure with aspect ratio 1:4 to see the impact of heat transfer and to decide the optimal shape that enhances the heat transfer mechanism. The benchmark quantities of average Nusselt number and kinetic energy values have been computed at various scales of the Prandtl number (*Pr* = 1,10), Richardson number ($0.1\le Ri\le 10)$, and Power law indices ($0.6\le n\le 1.4)$. The findings lead to the following conclusions:

- By incrementing in the value of *Pr*, the values of ${Nu}_{\text{avg}}$ increase.
- The value of ${Nu}_{\text{avg}}$ increases when the value of *Ri* varies from $0.1\text{ to }10$.
- Values of ${Nu}_{\text{avg}}$ and KE increases with the increase in the Power law index from $0.6\text{ to }1.4$
- It has been observed that the Circular heated obstacle had the highest values of ${Nu}_{\text{avg}}$ and KE as compared to the triangular or square heated block. Therefore, we can conclude that the circular shape is the most effective shape for heat transfer in this computational setup.
- The effect of *Pr* number is less significant at lower *Ri* values.
- The circular shape is superior to the other two in terms of heat transmission efficiency.

In future, this study can be extended to three‐dimensional flows involving shape and parametric optimization algorithms for the heated blocks.

## Data Availability

Data will be available on request by contacting the corresponding author, Dr. Ahmed Refaie Ali, via ahmed.refaie@science.menofia.edu.eg , OR via Dr. Afraz Hussain Majeed at afraz@ujs.edu.cn .

## List of symbols

*AR*: Aspect ratio
*C*_*p*_: Specific heat
*g*: Gravitational acceleration
*Gr*: Grashof number, dimensionless.
H: Height of the cavity
*K*: Thermal conductivity
L: Length of the cavity
n: Power‐law index, dimensionless
*N*_*u*_: Local Nusselt number, dimensionless
*p*: Pressure
$$\overline{P }$$: Dimensionless pressure
Pr: Prandtl number, dimensionless
Re: Reynolds number, dimensionless
Ri: Richardson number, dimensionless
*T*: Temperature
*U*_*lid*_: Velocity of the moving walls
*u, v*: Velocity in X and Y direction
$$\stackrel{-}{U,}$$$$\overline{V }$$: Dimensionless velocity in X and Y direction

## Greek symbols

$\beta$: Thermal expansion coefficient
$$\rho$$: Fluid density
$$\eta$$: Apparent viscosity
$\theta$: Normalized temperature, dimensionless
$$\mu$$: Dynamic viscosity
$$\upsilon$$: Kinematic viscosity

## Subscripts

*C*: Cold
*H*: Hot
